# Contagious Venereal Disease Amongst Horses in Kent County, Canada

**Published:** 1890-04

**Authors:** Peter H. Bryce


THE JOURNAL
OF
COMPARATIVE MEDICINE AND
VETERINARY ARCHIVES.

VOL. XI. APRIL, 1890. No. 4.

### CONTAGIOUS VENEREAL DISEASE AMONGST HORSES IN KENT COUNTY, ONTARIO.

By PETER H. BRYCE, M.A., M.D.

[CONCLUDED FROM PAGE 158.]

*Comparison with Venereal Disease elsewhere.*—Fortunately for the credit of British horses, so little of venereal disease has appeared amongst them that English veterinarians have been forced to seek in foreign literature, notably that of France, for descriptions of a disease which from time to time has appeared with great virulence in different sections of that country, and which created the necessity for government inspection and registration of stallions with a view to the eradication of the disease.

An extremely contagious disease affecting horses has been known in Germany, Southern Russia and Austria since the beginning of the present century, as in Pomerania in 1839, in Silesia in 1840 and in Wurtemberg in 1841. A similar disease has prevailed at times in different parts of Switzerland and France since 1830, notably in Algeria in 1847 and at Tarbes in 1851, and in a section of the State of Illinois since 1884. Of necessity, its literature, except of the outbreak in Illinois, belongs to a time when the biological features of disease had not been greatly studied, and the term *maladie de cöit*, applied to the disease in modern treatises, is very significant in its indefiniteness of the different views held regarding its real nature.

Among the terms which have been applied to it are *equine syphilis ; verole ; typhus venerien ; maladie pustuleuse* and *chancreuse maligne.* Such names have been applied to it by different observers, who, attracted by certain symptoms, varying in prominence by different outbreaks, have believed the disease to be a syphilis, a neurosis or nervous cachexia, a lymphangitis, a scrofulosis or nervous phthisis, or even typhoid fever.

Thus Fleming, in the 1875 edition of his classical work, "Veterinary Sanitary Science and Police," calls it an eminently virulent malady of a specific nature, generally marked at first by local signs and subsequently by constitutional derangement of a grave character. With the absence of personal knowledge of the disease and the collating of his materials from the writings of different observers of forty and fifty years ago, it is only natural that the views expressed by Fleming regarding the varieties should seem rather crude in the light of modern pathology. A purely arbitrary division of the disease into *benign* and *malignant* has been made by him, but nowhere except in the fact that some cases recover and some die, are there any phenomena belonging to the one which, except in the matter of degree of intensity, may not belong equally to the other. Thus each may begin with the mildest of local symptoms, nothing more than an excessive vaginal discharge and the persistence of œstrum being present, the mare being sent to the horse frequently. "This unusual excitement of the genital organs should arouse suspicion, if the disease is known in the country."—Fleming. Again he quotes Reynal as saying : "This benignant local form is accompanied by general symptoms somewhat resembling those of the malignant form, for it is to be remembered that between the two there are no sharply-defined limits, but rather an insensible transition according to the seriousness of the general or local symptoms."

It is also remarked that while the mild form usually gets well in a few weeks, yet it may last several months ; it may become exaggerated by repeated coition on the part of the mares, when the disease has been mistaken or unrecognized. It then becomes malignant. "In the stallion the disease is not so marked as in the mare, and not infrequently several weeks pass away without any indications of its existence being manifested. At other times it appears in a few days after coitus as an indolent, or œdematous, but intermittent inflammation of the prepuce, and in some cases

œdema with collapse of penis. The disease may become aggravated and malignant as in the mare."

Again speaking of the malignant form, Fleming says: "If recovery takes place, it is only in those cases in which the disease is little developed, the wasting stops, vigor returns, though there often remains paralysis of the labia of the vulva, and a thickened mucous membrane."

The disease may last from seven months up to two years; but frequently before this, intercurrent maladies cause death. Reynal remarks that the local symptoms are all that will be visible for as long as a year before other symptoms are exhibited.

Again the important statement is made that, "Toward the end of the covering season it may even seem to disappear altogether, but at the next season it will manifest itself with increased violence. In young and vigorous, well-kept horses, the malady may also continue localized for a considerable period; but in those which are weak or badly attended to, the general or secondary phenomena are not long in appearing. The disease may continue from three months to three, four or even five years."

But to further illustrate the ill-defined views which seem, at the time of the issue of Fleming's edition, to have existed with regard to equine venereal disease, we have treated, under another distinct heading, "Exanthemata of the Genital Organs," a disease which has been given the synonym *syphilis*, *pseudo syphilis*, *maladie aphtheuse des organes genitaux*. It is divided into three or four forms, viz.: Simple papules, vesicles, pustules or ulcers.

After stating that it is allied to gonorrhœa in man, Dayot's views of it, based upon inoculation experiments, are given, in which he describes the progress from millet-seed vesicles, an inflamed areola with serous fluid becoming purulent ulcers, even œdema of the vulva, confluent ulceration extending to vagina, rectum, perineum and mammæ. The inflammation may extend to the urethra, setting up urethritis, while in a few bad cases swelling and a lymphangitis of posterior limbs has been observed.

In most cases recovery takes place in a few weeks, but Latour has seen the disease last eight or ten months, and even have a fatal result. Lajoux says that in some cases there is a violent inflammation of the vagina with vesicles and ulcers; that the inflammation may extend to the mammæ, the vulva become[illegible] emphysematous as also the perineum and posterior limbs. Othe symptoms, such as denudation of pigment, shedding of pigment, violent pruritis, œdema of head and trunk with extreme urethritis and a hide-bound staring coat are described by Lafosse.

The disease greatly increases in extent and severity if a stallion is used. Treatment may not effect a complete cure, though the disease is rarely fatal.

We have abstracted at some length the salient points in these varieties of venereal disease as presented in the writings of standard authorities, and think that we have illustrated the fact that no greater differences, either as to manner of causation, variations in symptoms or their severity, have been shown to exist in equine venereal diseases, than have been shown to belong to gonorrhœa in man, with its chancroid manifestations—orchitis, buboes, synovitis, rheumatism, etc.

Inasmuch, however, as an outbreak of equine venereal disease in Illinois within the past six years has been carefully investigated, and as certain facts seem to create a suspicion of the outbreak in Kent and Lambton being related to it, if not directly caused by it, we may briefly refer to its character as set forth by W. L. Williams, V.S., employed by the State Board of Live Stock Commissioners to inquire into its nature.

From this report it appears "the disease was introduced from France by the imported brown 'Moore' horse at the age of three years, sold in the Spring of 1883 to Moore & Son, Clinton, Ill., and by them was placed in the stud near Clinton, serving a large number of mares besides those belonging to his owners, a fair percentage of which became impregnated and dropped healthy foals. In 1884 he was continued for a time in the stud without any complaint from patrons, but before the season was far advanced the horse became unwell. The genital organs became swollen and the penis sore in several places. At the ring of the penis (preputial ring) there was sufficient ulceration to destroy the ring at the front part. At the upper part of each flank there were extensive ulcerating sores, which healed tardily, leaving large cicatrices. On the left side of the crest of the neck there appears a very plain brand of the letters, 'D N,' which I am reliably informed by Prof. Law and Dr. P. Paquin, unmistakably indicates that this horse had been condemned by the French authorities for *maladie de cöit.* The horse was sold some time after the development of the symptoms of disease for a merely nominal sum, as a seriously diseased stallion, to his present owners, after having been taken from the stud and treated for his malady with indifferent results, and in 1886 and 1887 the horse's services were offered to owners of mares, but only a limited patronage was attained. Nothing particularly further was thought of the matter until the disease broke out in alarming proportions, and inquiry was instituted to determine its origin, when it was learned that in 1883 Milton Chapin had two black mares to the Moore horse, and disposed of one to Sandusky Wilson, and the two mares were bred in 1884 to 'Utopia'' before he was sold to Foley & Sennitt. It is now said that one or both of these mares were affected with equine syphilis when bred to 'Utopia,' and that they had contracted the disease from the Moore horse in 1883. Certain it is that the Wilson mare was bred to the Moore horse in 1883, and to Utopia in 1884, and that she was seriously diseased in 1884, undoubtedly of equine syphilis, partially recovered, was sold to a Mr. Yakle, of Waynesville, Ill., and by him to an unknown shipper.

"Returning then to Utopia, after having served the Chapin and Wilson mares, he was kept in stud by Harold & Culbertson, serving among others the 'Marvel' mare, and was sold apparently in March, 1885, to Foley & Sennitt, and proving unequal to the demand 'Black Diamond' was purchased of Harold & Culbertson to assist, and proving unsatisfactory was exchanged, June, 1886, for 'Perie.' During the Summer of 1885, Utopia served about sixty mares without, so far as learned, diseasing any, and in the fall of 1885 he served some twenty mares, of which a few proved in foal, while some fifteen of them became affected, nine of which have died and the others have most partially recovered, some of them showing scarcely any trace of the disease."

I have made these extended abstracts from the paper of Williams, in order that the method of propagation and distribution of the disease may be seen to have been the same in manner as that in Kent, and further for the purpose of showing that the essential characters of the disease are those not only as described by foreign observers, but those which we would expect to belong to a chronic urethral and vaginal catarrh, set up from whatever cause, either in man or horses, and perpetuated under exactly similar conditions.

After giving a synopsis of the literature of the subject Williams goes on to remark : "I am obliged to take exception to the generally accepted ideas of English writers and translators, and separate the so-called benign form from one of the diseases under consideration as a wholly distinct venereal disorder."

One of the facts upon which this reasoning is based is that he had not seen (no case of less than several weeks standing had been examined by him) and could not gather "from many intelligent horse-breeders who have had abundant opportunities for close observation that such eruptions have occurred to that marked extent described by Fleming and others in the present outbreak, and since Fleming, as previously quoted, describes these pustules and ulcers as occurring more particularly in this so-called benign form, we may well doubt, if not wholly deny, the occurrence to any marked extent of any such eruptions in the affection under consideration, especially when we remember that such do occur in abundance in the benign venereal disease, with which English authors so hopelessly mix the true equine syphilis."

We cannot but come to the conclusion that while Williams is quite right in the statement that the English writers "hopelessly mix" the benign and malignant forms of the disease, he must surely have forgotten the description, already quoted, which he has given of the "Moore" horse, the supposed origin of the disease, when he makes the absence of ulceration a point for differential diagnosis. He describes the denudation of pigment, both in stallions and mares, a condition by other writers due to the acrid discharges from a pus-secreting mucous membrane. He further points out the presence at times of swelling of the vulva, extending along the perineum to the mammæ, and states that the vaginal glands and even the mammæ may suppurate. To make the description of pus absorption complete, it is further stated : "There occurred frequently, suddenly appearing, sharply defined circular or various shaped flattened swellings," and again : "The elliptical welt-like swellings of the skin are usually also more marked in the stallion than in the mare. There is also a strong tendency to suppuration of the vaginal and scrotal lymphatic glands. The lymphatics of the sheath and scrotum were still more prone to suppuration."

Nothing further than these quotations is needed to show how slight are the grounds upon which Mr. Williams divides the outbreaks which he has investigated into distinct diseases. But to fully illustrate the point that the milder cases in which eczematous eruptions occur are part and parcel of the same cause and the occasion of the more serious condition, I shall quote from the recent remarkably concise and scientific article by W. T. Alexander, M.D., of New York, on "Diseases of the Lymphatic Glands." He says: "Suppurative inflammations which give rise to glandular swellings may conveniently be divided into two classes, superficial and deep. Of the former are inflammations of the skin, and catarrhal affections of mucous membranes; of the latter, subcutaneous, phlegmonous, local and diffuse furuncles, onychias, burns and frost-bites, and all gangrenous and deep ulcerative affections. Catarrh of the gastro-intestinal and pulmonary mucous membranes are the most common causes of affections of the former class. Here the lymph follicles seated in the mucous membranes are first affected, and later the glands connected with them. Thus a very close relationship is noticeable between diseases of the bronchial tubes and their lymphatic glands. The affections of the glands here vary, with the duration and intensity of the bronchial affection, from simple hyperæmia or hyperæsthesia to chronic inflammation, with pigmentation, cheesy degeneration or calcification.

"In such cases the most plausible explanation of the adenitis is that the delicacy of the inflamed epithelial stratum affords no obstacle to the passage to the deeper layers of the membrane of the noxious organisms always present in the inspired air, or the contents of the intestinal canal, as the case may be. Similar affections prevail in the superficial affections of the skin, and in certain forms of eczema, which disease has been regarded by some as the analogue of a catarrh of the mucous membrane.

"In these cases the resisting or protective power of the epithelial layer is much lessened or entirely overcome, and in consequence of this the integrity of the *rete malphigii* is impaired, and the passage of irritating organisms into the subcutaneous tissue permitted. For this reason there are but few cases of chronic eczema which are free from sympathetic affections of glands. In fact, swelling of these organs is a necessary attendant upon all itching, secreting and most scaly diseases of the skin, including the prurigo of Hebra, scabies and phthiriasis capitis. In all these instances, the cause of the swelling is doubtless the same, viz., the penetration into the subcutaneous tissues of noxious agents, deposited on the surface (access being gained through the lesions of the skin produced by the fingers of the patients), and then transportation into the lymphatic vessels and thence into the glands.

"Having reached these organs the foreign organisms act as irritants, causing a swelling of the glands, which stand in an exact relationship with the specificity and intensity of the original local process. If the original lesion be, for instance, a *soft chancre*, an acute suppurative adenitis is developed, which may be carious or gangrenous, if the chancroid itself take on these changes. If connected with more indolent or less virulent lesions, the affection of the glands is also sluggish and indolent.

"In the case of a circumscribed affection of the skin, *or of a mucous membrane*, the swelling is for a time confined to a few glands, but later on in the course of the disease the entire groups in connection with the affected region become involved. If recovery do not take place, the swelling next invades the next group of glands in the direction of the lymph current."

Evidently, therefore, the intensity of the local infection, assuming the microbe which is the origin of the disease, in any given case to be the same, is dependent upon (*a*) the amount of virus, *i. e.*, the number of microbes absorbed; (*b*) the specific virulence of the inoculating medium; (*c*) the favorable condition or the resisting power of the system of any animal to the disease at the moment of its inoculation. Pasteur, Davaine, and many other experimenters have made this plain. M. Roux admirably illustrates this by the influence which oxygen exerts on the virus of chicken cholera at a temperature of 33°C. No attenuation of the virus will have taken place in the first week. One drop inoculated causes the death of any fowl; yet at the end of two months the same virus will not kill at all. The harmlessness of the virus exposed to oxygen can be perpetuated for successive generations. Says M. Roux: "By this experiment M. Pasteur established the attenuating influence which the air possesses; at the same time he explained how it is that the activity of a virus, under natural conditions as seen in epidemics, is preserved or exhausted, and how the same malady may be sometimes malignant, sometimes light."

Remembering these now established facts, it becomes possible for us to reconcile the otherwise confusing statements made by different observers of the disease under consideration ; and when we think of the variations in individuals and breeds of horses, the different climatic conditions and varying seasons in which these outbreaks have been observed, the varying degrees of exposure to the disease, and of the care in feeding and cleanliness observed by different horsemen, it would indeed be strange, from the knowledge we have of other diseases, if just such differences as have occurred in France, in Illinois and in Kent had not occurred.

*Extent of the Disease in the Kent District.*—The history of the progress of the disease in Illinois, as already given, is admirably illustrative of the method of its propagation in Kent. From whatever first source, it is certain that in 1887, in the Ridgetown district, some several stallions became affected with the disease, as may be gathered from the evidence of W. B. Rowe, V.S., and that of G. Murray, V.S. Following the history of the disease in Illinois, it seemed less prevalent in 1888, but has somewhat increased again in 1889. Other instances appear showing that a venereal disease, contagious and with similar symptoms, had occasionally been seen by horsemen in stallions as much as fifteen years ago. In 1888, the stallion "Henry Abraham," a Percheron imported from Michigan in 1886 by Richard D. Courtney, of Moore township, Lambton County, was the cause of the outbreak in the Chatham district. The history connected with this horse is an interesting and important one. Courtney stated that he purchased the horse in Marine City, Michigan, to which place he had been brought in 1884, from the stud of Stubblefield & Armstrong, Bloomington Co., Illinois. Although there is no history of the existence of any venereal disease in this stud, yet a remarkable statement is found in a letter written by Mr. G. W. Stubblefield in answer to inquiries made regarding "Henry Abraham." It is stated therein that "Henry Abraham," 2d (693 of the Percheron Stud Book), bred by him, was dead "some four years ago." As this is the period, according to Courtney's statement, at which "Henry Abraham" was bought from Stubblefield & Armstrong and taken to Marine City, there seems only one inference to be derived from Stubblefield's statement, viz., that for some reason best known to himself he wished "Henry Abraham" to be considered as dead. As at this time he was only four or five years old, and is said to have been a very fine looking animal, it seems strange that if there was nothing wrong with him a stallion of his breeding should have been *lost sight* of. If it be true that this horse was diseased in 1884, and begat healthy colts for three years thereafter, infecting, as far as I could learn, no mares before 1888, it would illustrate not only the mild character which this disease may retain as regards the infected animal for many years, but also how a once affected part may light up again an inflammatory action which may become contagious. Such views, however, are held by prominent veterinarians, amongst which may be that expressed by F. Grenside, V.S., of the Ontario Agricultural College, regarding urethritis, as also that of Prof. Andrew Smith, Ontario Veterinary College. Another very reasonable explanation, however, of the source of the disease in this horse is that found in the evidence of M. McGregor Courtright, going to show that it is probable that the Percheron horse belonging to J. Smith, of St. Clair, Michigan, had infected some mare subsequently bred to "Henry Abraham."

The most important source of the extended outbreak of 1889, which has been the occasion of this Commission of Inquiry, is the horse "Fairfield Chief," owned by Frank Conduit, of Tilbury Centre. A reference to the evidence of Mr. Conduit, in which the history of this stallion is given, is both interesting and important. I think it may fairly be concluded that—in the absence of all evidence to the contrary—this stallion had not been affected before the beginning of the season of 1889. A possible source of the disease is to be found in the mare belonging to John O'Neil. She had been bred the previous season, but did not get in foal. The excitement of œstrum, if Conduit's statement be true, remained present to such an extent as makes one suspect that a chronic inflammation continuing from the previous year had been present, and that "Fairfield Chief," without being personally affected, at first at any rate, became the carrier of the disease to those mares subsequently infected by him. We have seen that this was the way in which William White's mare was infected by "Young Hawthorne" two days after he had covered a mare, thought at the time to have recovered from the disease. Indeed, it seems the most natural thing that where a horse, after covering an infected mare has not at once been carefully cleansed, he should retain the infection within the sheath, and hence continue to be the occasion of spreading the disease while perfectly free from it himself. That he should, however, likewise become infected is most probable. The opinion expressed by Dr. Samson, of Blenheim, to the effect that such infected horses are likely to become chronically diseased, in the same way and even more so than men, seems wholly borne out by many facts.

*C. Recommendations deemed necessary for dealing with the Disease.*

In view of the biological and clinical facts which have been set forth in the two preceding sections regarding the outbreaks of venereal disease which, as the exhibit above given of animals infected shows, has prevailed at different places in the Western parts of the Province during the past three seasons ; regarding the extremely contagious, yet insidious nature of the malady, no matter what its pathological relationships may finally prove to be ; recognizing that, no matter how mild or how severe its effects on the stallion or mare may be, its history is that of a disease which like other catarrhs is peculiarly prone to become chronic, retaining its infective properties whenever localized hyperæmia, due to œstrum in the mare or over use of the stallion, is set up, or when excessive secretion of the vaginal mucous membrane, in consequence of a reduced tone of the general system, is present, and finally remembering the numerous avenues through which the dissemination of the disease may take place, due to—

(*a*) Limited symptoms of constitutional disturbance and of outward signs ;

(*b*) Ignorance on the part of owners of mares and the grooms in charge of stallions as to the meaning of those symptoms and signs which do appear ;

(*c*) To the universal tendency which exists in the matter of disease whether of man or the animals belonging to him, to overlook everything which does not early develop serious results ;

(*d*) To the selfishness and dishonesty of others who for a temporary material advantage might expose mares to a stallion, or a stallion to a mare, when the disappearance of outward signs makes an animal appear free from the disease ; and

(*e*) To the commerce which is continually going on by which animals are traded to persons in neighboring districts or sold to other parts of the country or to foreign countries, the interest of individuals, of the proprietors of large stock farms and the good name of Canadian horses, all demand that the most serious attention be paid to the questions of: (1) Whether any regulations should be made with a view to limit the existence and prevent the spread of the disease; and (2) if so, what should be the essential character of said regulations? After a careful review of all the sworn evidence of competent witnesses, as veterinarians, large stock owners and breeders, after a personal observation of the disease and a careful review of all the literature of the subject at my disposal, I think there can be no doubt, not only as to the necessity for, but also to the expediency of, statutory powers being given to make regulations with a view to limiting the spread of this disease by stamping out in every possible way the contagion where now existing within the Province and by preventing by such measures as are practicable its introduction into the Province from other countries.

The measures to be taken with reference to the disease are naturally divided into *first*, prophylactic regulations applicable to contagious diseases generally; and *second*, to those necessary for stamping out venereal disease when actually present in a district or in the whole Province.

### 1. Prophylactic Regulations.

These have to be considered with reference, (*a*) to preventing the outbreak of contagious diseases generally; (*b*) to preventing the exposure of healthy animals in districts where venereal disease is present.

(*a*) *Prophylaxis against Contagious Diseases in General.*—Fortunately the Province has experienced in the past very little danger from equine contagious diseases. Glanders has from time to time made its appearance in sporadic form, and occasionally it has demanded a special inspection in localities where it has been to some extent prevalent. An efficient Provincial statute is already in force, while municipal powers under the Public Health Act are given for dealing with all cases of it known to exist. The fact of its being a lingering disease, by which animals may be capable of working for months, during all which time they are dangerous from their power to communicate it, makes the question of whether some compensation to owners for giving early notice of its existence in their animals, which the law requires to be destroyed, ought not, in the interest of public health, both of men and horses, to be considered. Epizootic influenza, from which Canadian horses have at times severely suffered, demands serious attention. Its extremely contagious character from contact makes prompt isolation of affected animals a *sine quâ non* to its limitation. This isolation should mean that an infected animal be removed from any stable where other horses are present, and the stalls where they have stood thoroughly disinfected by corrosive sublimate or carbolic acid solutions, thus destroying the discharges from nostrils, etc. The ordinary practice of common stabling in large studs, livery stables, hotel stables, etc., is fraught with the greatest dangers, since mangers, drinking troughs, etc., are all liable to harbor the infection. Penalties for bringing affected animals to public stables ought to be stringently enforced.

Reverting to venereal disease, it may be said that its at present localized existence, and the fact that it can be communicated solely by coition (except where, as in the case of foals, the discharges from the mare are inoculated into the eyes, by switching the tail, etc.), make it a much easier matter to prevent its outbreak than in the case with such diseases as influenza. Such prophylaxis, however, demands the initiation of a system which, though in vogue in foreign countries, has never been introduced into Canada. This is the registration of all stallions intended for breeding purposes, whether imported or native-bred. The appended evidence of extensive breeders, and of several veterinarians, bears very strongly on this point. This evidence goes to show that it is most desirable for the improvement of Canadian horses that stallions of superior strains of blood should alone be used. From this it follows that a registration, based upon a thorough inspection of all stallions for public service by competent veterinarians, be instituted by Provincial regulation. Without discussing this point, it being foreign to our subject, the same evidence bears equally forcibly upon the necessity for registration of all stallions, based upon an inspection as to freedom from venereal disease. The reasons why this is so necessary have, in the two preceding divisions of the subject in which the insidious and persistent characters of such diseases have been discussed, been fully set forth. We have seen how the importation of foreign stallions was the occasion of its appearance in France, of its introduction into Illinois, and of how a strong suspicion exists of its introduction in two instances into western Ontario from two stallions from the latter State. Manifestly, therefore, a period of quarantine similar to that at present exercised in the matter of imported cattle is desirable, during which period inspection of stallions and of mares, imported or native, bred to them during this period of observation ought to be carried out. An affidavit giving the history of such stallions for at least a year preceding importation ought further to be required. As to native-bred stallions it might be said that the same reasons for inspection do not exist. Were no disease already in the Province this could be argued with much reason. But with disease having existed in the Province to the extent that is set forth in the preceding section, it is apparent that we have present the same reason for inspection and regulation of these latter as we have in the case of imported stallions ; since it must be remembered that a constant commerce goes on in horses, by which animals are transported from one part of the Province to another. This may be expected to be especially the case with stallions which, for whatever reason, have obtained a bad reputation in any district. As pointed out in evidence it is a common thing for a stallion to go under one name in a district, and thereafter, when transferred to another district, to have another name given to him. Provincial registration would make this impossible. In the case of stallions infected in the Chatham district we may naturally expect that this will take place before next breeding season, and I have been informed, on good authority, that the trading and sale of mares which were infected during the past season has been steadily going on. Provincial registration under this aspect of affairs becomes an apparent necessity.

(*b*) *Prophylaxis in those Districts where Venereal Disease Exists.*—This must extend both to mares and stallions. In districts such as that of Chatham, all stallions having a history of having been affected during past seasons must be first considered. Evidence from every source goes to show that while such stallions *may have been cured*, yet they must be viewed with the greatest suspicion. The closest inspection must, therefore, be made of them, and if they appear perfectly sound, then if allowed to do public service, their health and that of every mare bred to them, must, during the whole season, be most carefully inquired into, for a time weekly, and if nothing unfavorable during the first month appears the inspections might then be made less frequently.

Mares having a history of previous infection are equally or even more to be dreaded. Careful examination of them before being covered is an absolute necessity, since one contaminated mare may, as we have seen, become the occasion of lighting up the disease in a stallion, to be spread by him along his whole route. Prompt notification by the owner of any animal becoming infected, under severe penalty for default, must be absolutely insisted upon.

To avoid contamination from unknown sources, the groom ought to be specially warned by the inspector to preserve the most rigid rules of cleanliness and disinfection. With reference to the stallion, washing all exposed parts after each copulation may fairly be insisted on ; while a less frequent daily use of the horse than is practised by the owners of second-class horses ought to be encouraged.

Too much cannot be said against the wholly unnatural, filthy and dangerous practice of allowing mares to go to the stallion at the period of first œstrum, usually within twelve days after delivery of a foal. Lacerated, congested parts are the most favorable condition for inoculation, while discharges which frequently are of a purulent character become, we believe, a not unfrequent cause of contamination in the stallion. The practice ought to be strictly forbidden, as is pointed out by some of the most observant breeders.

### 2. Measures for the Suppression of Existing Outbreaks.

The history of venereal disease in the Chatham district is not encouraging, all evidence going to show that it has been increasing during the several past years. Provincial and local interests alike demand its speedy suppression. The quotations already given have shown how gradually the disease extended in Illinois, and how, when its nature was recognized, the live stock commissioners empowered the destruction with compensation of all animals proved to have been once infected. Undoubtedly this is, though radical, the most efficient means of ridding the district and Province of the dregs of the disease. But should the malignity of the disease seem not to be such as to demand such radical measures on the part of authorities, then the adoption of provisional measures, such as those indicated in the following extract from Fleming, ought to be rigorously enforced :

*Provisional Measures.*—As the disease (*maladie de cöit*) is propagated solely by sexual intercourse, when it prevails in a country the only provisional sanitary measures necessary are those which will prevent diseased animals from being employed for breeding purposes ; at the same time giving notice of the existence of the disease and the precautions to be adopted by those whose interests may be endangered. Above all, should the disease be suspected in any animal, the owner, or whoever else is responsible, ought to be compelled to report the circumstance to the proper authorities, as in the case of other contagious maladies.

In Austria, where the contagion has prevailed somewhat extensively for many years, the following regulations are in force :

"1. Even when there is nothing to lead to the suspicion that the disease exists, every mare which is put to the horse shall be inspected in the presence of the principal communal authority ; and a rigorous refusal shall be given to all which are too old, or in a weakly condition, or which have a discharge from the vulva in any way different from that observed in mares only during œstrum.

"2. The penis of the stallion intended to be employed for such purpose shall be examined at different times, and if it offers any lesion, no matter of what description—even if it has nothing in common with those of the disease in question—the animal must be excluded from use until perfectly cured.

"3. There is every reason to make known to the breeders by the most practical means the characters of this disease, so that they may be able to recognize it at its commencement.

"4. If a breeding stallion is suspected, the owner, through the medium of the local authority, shall immediately make it known to the district administration, who will, without delay, order an inquiry to be directed into the circumstance, and prescribe the necessary measures.

"5. In order to prevent the extension of the disease to other districts, the sale of breeding-horses shall be interdicted in the infected district during the existence of the disease.

"6. If the malady has acquired a great extension in a country the employment of all stallions for breeding purposes, whether they belong to individuals or to the State, shall be suspended. If any owner infringes this order, he shall be made liable to the penalties laid down in the Austrian penal code. The stallions recognized as diseased, if they belong to private persons, shall be sequestrated and submitted to proper treatment under the surveillance of the police.

"7. Horses affected with the disease shall be separated from the healthy, and attended to by persons specially selected for the purpose; the necessary utensils shall not be used with other animals; and if the diseased are not deemed incurable, they will be submitted to treatment.

"8. In order to arrive at a knowledge of the exact state of the epizooty, every eight days at least there should be a general revision of the equine population in the infected locality.

"9. The horses attacked by the disease should be castrated, as well as those which, notwithstanding their apparent good health, have transmitted it to mares they have served; also those which have been put to mares affected with the malady when they were so served. The epizooty commission shall decide, without right of appeal, if it is necessary to submit a stallion to this operation.

"10. The mares affected with the malady in its benignant form should not be allowed, even in the following year, to be served until after they have been inspected and declared healthy by a veterinary surgeon. The mares which have the disease in a malignant form shall, if they recover, be excluded from breeding, and shall be branded on the left side of the neck with a hot iron.

"11. If the disease be complicated with glanders or farcy, the animals shall be submitted to the measures in operation against these affections.

"12. The carcasses of horses which have died or been destroyed because of the malady, as well as the infected stables and utensils, shall be treated as prescribed for glanders."

In Prussia, according to a ministerial decree of September, 1840, various measures are laid down for the suppression of the malady. Chief among these is that relating to the declaration to the authorities of its existence in stallions, and putting a special mark on those which are affected, in order that they may be recognized. They are not allowed to be removed from the locality within three years after their recovery. When the disease prevails in a district its existence is made known by an official publication; after the issue of which no stallion is allowed to serve mares unless its owner is provided with a certificate of health furnished by a veterinary surgeon, and not dating earlier than fourteen days. It is the same for mares: though their certificate should not be for a longer date than four days.

Were such regulations in force and provision made for their efficient enforcement, this, as other diseases of the zymotic class, would very soon disappear or become so limited as to be no longer a cause of alarm.

It only remains for me to indicate what would seem to be the most practicable system of inspection and registration. As in other matters we have federal, provincial and municipal authorities, all or any of which may be clothed with powers to enforce any regulations based upon statute. The question of: What system would likely prove most efficient? was asked of different witnesses and almost without exception the view was held that governmental appointment of inspectors would probably be the best, as their appointment and action would be largely free from the pressure of local influence. We thus have the question reduced to that of whether the work should be done by the federal or by the provincial government. At present the Canada statutes, 1886, Chap. 69, deal only with horses at ports of entry. This Chap. 69, Sec. 27, empowers the Governor in Council to make any necessary regulations for subjecting foreign horses to quarantine, and for regulating their importation into Canada. Similarly, Sec. 39 makes provision whereby horses imported or introduced contrary to any regulations relating thereto in force shall be forfeited and offenders subjected to penalty as provided by law. Manifestly, therefore, we have a mode by which the introduction of the disease into Canada can be prevented. What now is needed is statutory powers for internal regulations. This, by a letter of the Canada Department of Agriculture to your Department, dated August 3d, 1889, is relegated to the provincial Governments.

The basis upon which such an Act may proceed, if introduced into the Legislature, would seem to be on the assumption that a uniform system of regulation be framed under it which would be applicable to any county, district, or to the whole Province. Under such, the Department of Agriculture for the Province could appoint veterinary inspectors for such areas as might be found most convenient for carrying out the work, arranging a scale of fees for inspection and license, to be levied on owners of horses at time of registration, and paid in either to a district official, *e. g.*, a county treasurer, or the Department of Agriculture.

A board of live stock commissioners might be appointed for the Province, as is done in various Western States of the Union, with functions similar to theirs. Inasmuch as local prophylaxis in order to be thorough must depend upon continual watchfulness, I can think of no system which can conduce to this end equal to that by which local Boards of Health (as is required by statute in Michigan). which in townships are made up usually of the more intelligent and influential agriculturists, be empowered to inspect through their sanitary inspector (who may be appointed as being specially qualified, as in the case of Chatham Township recently in the matter of hog cholera), and to report suspected cases to the distiict inspectors and, where ordered, maintain local supervision and isolate and disinfect, as the case may demand. The basis upon which payment for inspectors should be made is a matter of detail which does not come within the scope of this report.

